# Experimental Analysis of Mechanical Anisotropy of Selected Roofing Felts

**DOI:** 10.3390/ma14226907

**Published:** 2021-11-16

**Authors:** Bartosz Łuczak, Wojciech Sumelka, Artur Wypych

**Affiliations:** 1 Institute of Structural Analysis, Poznan University of Technology, Piotrowo 5, 60-965 Poznań, Poland; 2 Institute of Materials Engineering, Poznan University of Technology, Jana Pawła II 24, 60-965 Poznań, Poland; artur.wypych@put.poznan.pl

**Keywords:** roofing felt, mechanical material anisotropy, microstructure of roofing felts, chemical composition of roofing felts

## Abstract

In this work, four representatives of roofing felts are under consideration. Special attention is paid to the mechanical behaviour under the tensile load of the samples. The results of strength tests for the entire range of material work, from the first load to sample breaking, are shown with respect to a specific direction of sample cutting. Moreover, a unique study of the microstructure obtained with the scanning electron microscope and chemical composition determined by energy dispersive spectroscopy of the tested materials is presented. The significant mechanical material anisotropy is reported and moreover argued by microstructure characteristics. In perspective, the outcomes can give comprehensive knowledge on optimal usage of roofing felt and proper mathematical modelling.

## 1. Introduction

Due to its low cost and relatively simple installation, roofing felt is a well-known material for decades and is still widely used as waterproof insulation in commercial and residential buildings. They are available in many types and variants, differing in purpose, thickness, materials or finishing. In this article, we will focus on a selected representative of this group. Despite this popularity, there is not much research on these materials (mechanical) properties nowadays.

Several main research areas can be distinguished for roofing felt, general information and advice. In this category, discussion on installation methods and practical characteristics of modified bitumen is presented in [1], overview of available modified bitumen materials and systems are presented in a [2], whereas [3] investigates the impact of application method on the ability to make a connection to provide proper waterproofing and strength.

Material modification through the use of various types of admixtures can be found in [4,5,6] where very interesting investigation of modification of the bitumen with a combination of liquid rubber is presented. In [7] roofing shingles are considered for which, however the base material is the same, but replacements for atactic-polypropylene (APP) and styrene-butadiene-styrene (SBS) are suggested. A study comparing roofing membranes constructed with standard and styrene-ethylene-butylene-styrene (SEBS) modified bitumens are discussed in [8]. Another work [9] describes a way to change bitumen with polymers to produce a membrane that can be applied using either an adhesive or hot-air welding process. Roofing material are analysed in more detail in papers by McNally and Fawcett [10,11], which contain molecular properties of bitumen-polymers blends and discuss how these properties impacts analysed material, making them suitable for the production of built-up roofing membranes. Zielinski in the article [12] turns to focus on the impact of the amount of SBA modification in bitumen on resistance of the material to low temperatures. On the other hand the subject of mechanical and chemical changes as a result of heat was taken up by Liu [13].

In recent years, in connection with the more and more frequently raised ecological factors, as well as the growing interest in the impact of those materials on the environment and how to recycle them, a lot of attention has been given to these environmental issues. The influence of roofing material on concentrations of pollutants in roof runoff waters and how they are concentrating time were investigated in [14]. Another ecological aspect is the estimation of the greenhouse gas emissions during the production of roofing materials these aspects are presented in [15] for bitumen modified atactic polypropylene (APP). Another rarely discussed way to reduce energy consumption is presented in [16], where a significant impact of the roofing material on the total energy consumption is noticed. An attempt to find the most practical solutions in economic and energetic terms is presented in [17].

Interesting research is also being carried out in the field of roofing felt material life expectancy assessment and fatigue. A service life prediction approach for roofing membranes abased on probabilistic modelling is presented in [18]. The issues of the destruction of the roof structure and its surface layer are extensively discussed in [19], and static, and cyclic fatigue were the subject of research [20]. Moreover, related research on material weathering and ageing which significantly affects the behaviour of the materials. Work [21] presents results of natural weathering and testing in a hot-dry climate, and in [22] effects of ageing on the change of material properties at low temperatures are investigated and aeat-ageing is also the subject of research in [23].

Another group of studies that can be distinguished are studies comparing roofing felt materials with each other and addressing the issue of material properties in various conditions. Mayer [24] presents a comparison of the low pitched roofing materials. Oba and Hugener [25] elaborate characterise polymer modified bituminous roofing materials. Furthermore in [26] different roofing materials are categorised, according to their capacity to reduce urban temperatures. Assessment of roof membranes in terms of extreme weather conditions, in this case hailstorm is presented in [27].

The last group worth mentioning are the strength assessments and numerical analyses of roof membranes. Results for biaxial load-strain testing of selected roofing sheet materials are presented in [28]. Dupuis in [29] shows load-strain properties and watertight/strain levels of aged polyester-reinforced roofing membrane. Results of long term evaluation on selected modified bitumen roofing systems could be found in [30]. Dynamic effects of wind on roofing systems are analysed in detail in [31,32,33]. Moreover, the impact of various thermal conditions on installed roofing membrane are presented in [34]. Finally, finite element analyses of roofing systems are presented in [35,36,37,38] where the mechanical performance of built-up roofing membranes, fully bonded to an underlying deck or substrate, was discussed.

In this work, we present the results of strength tests for the entire range of material work, from the first load to sample breaking. Moreover, the study shows pictures of the microstructure and chemical composition of the tested materials. Finally, material anisotropy (argued by microstructure characteristics) is studied in detail, giving comprehensive knowledge on more optimal usage of roofing felt.

## 2. Methodology

The subject of this research is to determine the tensile strength of roofing felt in the entire range of its work, namely elastoplastic up to damage. Four different types of roofing felt were analysed, differing in thickness, base material and surface finishing methods. In this paper, the signature of these materials are introduced—M1, M2, M3, M4. For three out of four tested types of materials, the matrix was made from the same material, and this material was non-woven polyester (materials M1, M2 and M3). The matrix was made of a glass veil in the fourth of the analysed material (M4). Two out of four types were made of elastomer-modified bitumens (M1, M3), one of them made of modified bitumen synthetic rubber (M2) and one was made from oxidised bitumen (M4). The thickness of analysed products was 2.2 mm (M2, M4) and 5.2 mm (M1, M3). The given thickness for all samples was within the deviation declared by the manufacturer for M1 and M3 material, it was ±0.5 mm, and for material M2 and M4 it was ±0.4 mm.

**Figure 1:**
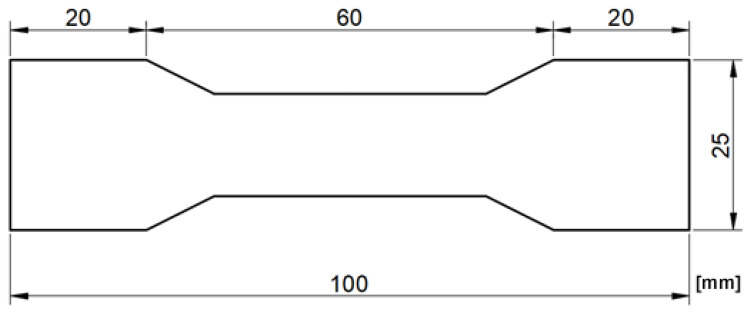
Geometry of testes samples.

**Figure 2:**
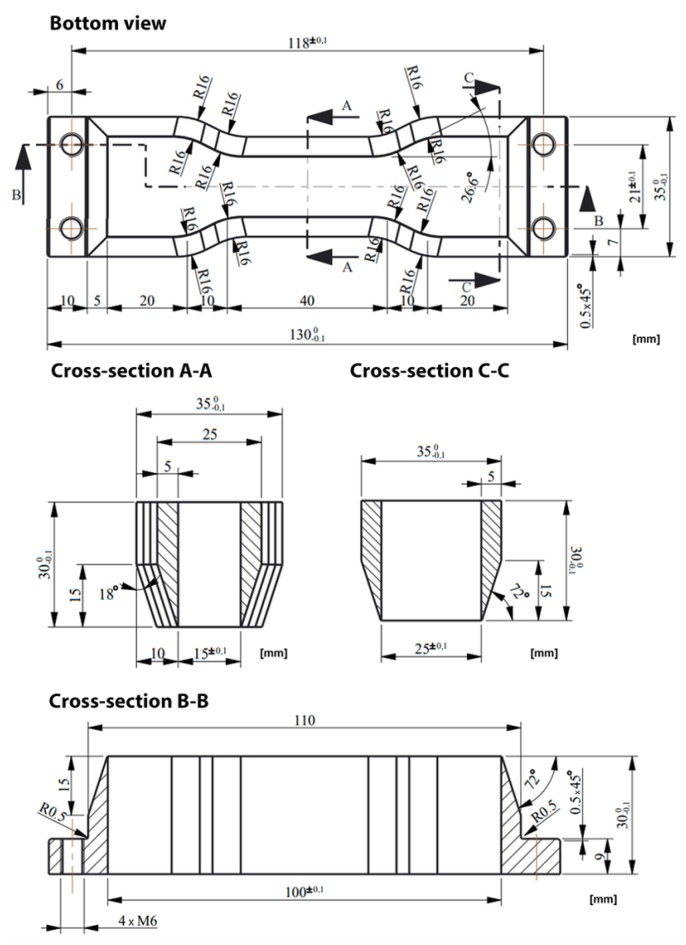
Cutting knife geometry.

The sizes of the testes samples were the same and are presented in Figure 1. To ensure the same dimension of the samples, a special cutting knife was prepared—this tool is shown in Figure 2. Samples were cut from sheets at least 100 mm from each edge to limit the influence of the edge zones of the sheet. Around each specimen, there were left a 50 mm space to minimise any influence of the sample cutting process.

**Figure 3:**
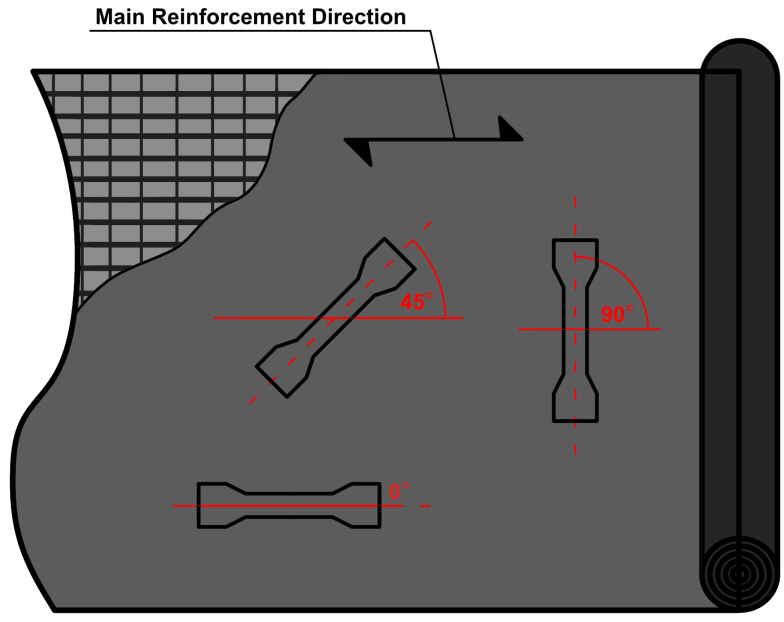
The direction in which the specimens were cut from the sheet (solid red lines represent the main direction of reinforcement).

Specimens were cut from the sheets at three different angles to the direction of the material reinforcing fibres. These angels are 0° in this case fibre runs parallel to the loading direction, 45° and 90° in which fibres run perpendicular to loading direction—cf. Figure 3. From that moment in work, the sample material and the angle at which the sample is cut in relation to the sheet matrix will be marked with a capital letter M; then the material number will be given (1–4), then the angle of cutting the sample from the sheet will be given in brackets, e.g., M1(45°), means material M1 cut in 45° angle with respect to the direction of reinforcing fibres.

## 3. Description of the Experimental Procedures

As it was described in the previous section, the studies were divided into three series differing in the angle at which the samples were cut in relation to reinforcing fibres. The single series consists of 10 samples of each material, and in three directions. There were 30 samples of each type of material, which gave a total of 120 samples tested.

All experiments were carried out at room temperature. Before tests, cut samples were stored in dry and dark conditions also at room temperature.

**Figure 4:**
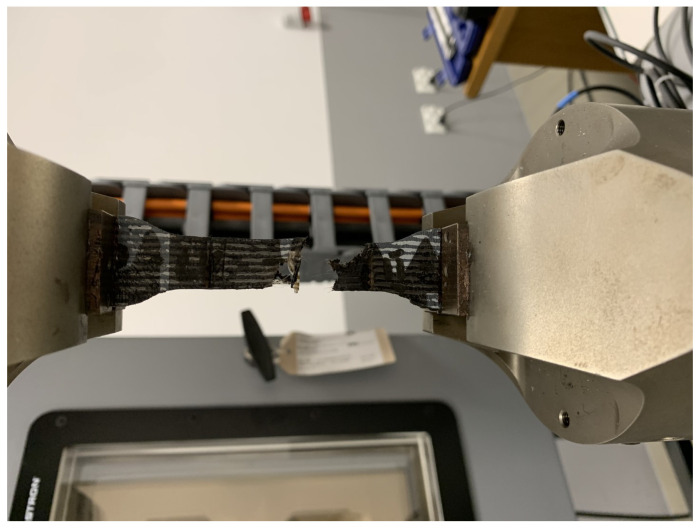
Sample after the testing procedure.

Distance between the upper and lower jaws of the testing machine was constant and equal to 70 mm, and this value is the reference length in the calculation. The strength test was carried out with a constant speed of deformation equal to 100 mm/min, until the sample breaks or there is a sudden drop in force value—Figure 4.

During the experiments, test time, the actual force and actual displacement of jaws were recorded.

## 4. Microscopic Structure and Chemical Composition

### 4.1. Scanning Electron Microscope Results

This section presents results taken with the scanning electron microscope Tescan Mira 3. They are divided into four groups depending on the material of the samples. Pictures were taken with a scanning electron microscope at different magnifications—10 µm, 50 µm, 200 µm, 500 µm (a scale of a photo is presented in each picture). The photos show the surface of the cross-section of the samples also taken at the highest possible magnification, which was limited by the type of material. These photos are unique because they present the structure of the material and because they were made without spraying with conductive coatings. In consequence, direct observation of the tested preparation was possible.

**Figure 5:**
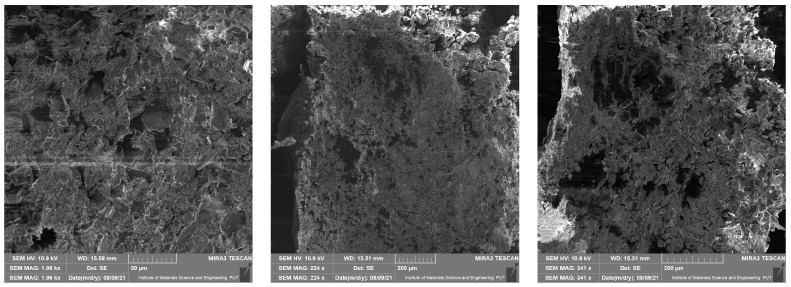
SEM photos for material M1.

**Figure 6:**
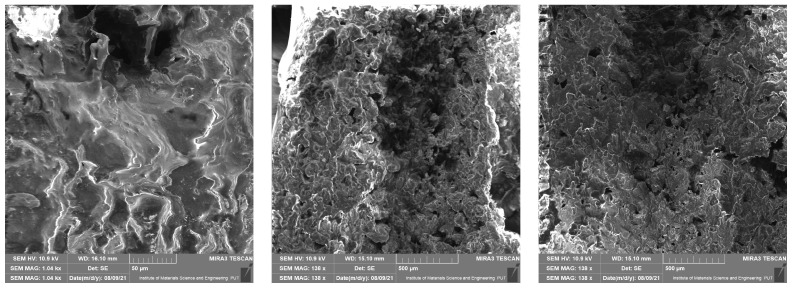
SEM photos for material M2.

**Figure 7:**
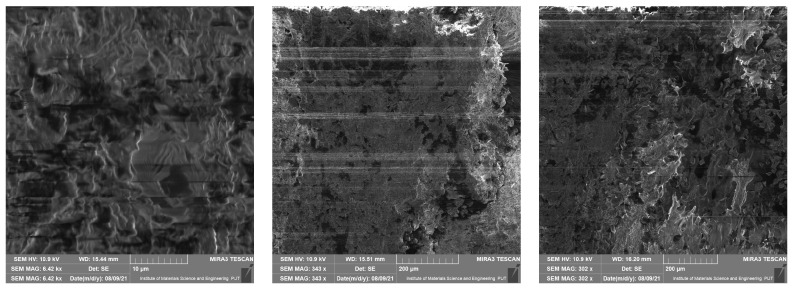
SEM photos for material M3.

**Figure 8:**
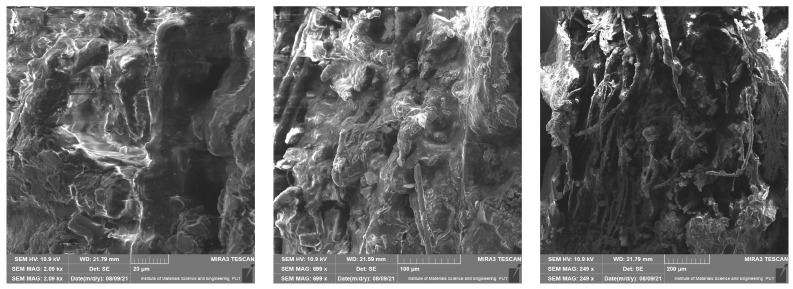
SEM photos for material M4.

In Figure 5, Figure 6, Figure 7 and Figure 8 it is shown that tested materials have a heterogeneous nature. Voids, grains of unequal size, and in one case, grains elongated in one direction can be observed. As a result, during mechanical testing, different strengths of bonding between the grains and different strengths of bonding the grains with the matrix material were captured (cf. Section 3). As mentioned, the magnitude of the magnification varies from 10 micrometres to 500 micrometres and results from the objective, most legible representation of the image (Figure 5, Figure 6, Figure 7 and Figure 8). The structures of all the felt samples show porosity. At the same time, the shape and arrangement of these pores are characterised by isotropy in M1–M3 materials. Due to the visible anisotropy, the M4 material is characterised by a lamalar pore distribution (Figure 5, Figure 6, Figure 7 and Figure 8). Therefore, an anisotropy of mechanical properties in this material is to be expected, as demonstrated in the part of this work describing the mechanical properties of materials.

All of these factors significantly affect the final strength of the material and induced considerable mechanical anisotropy.

### 4.2. Energy Dispersive Spectroscopy

**Figure 9:**
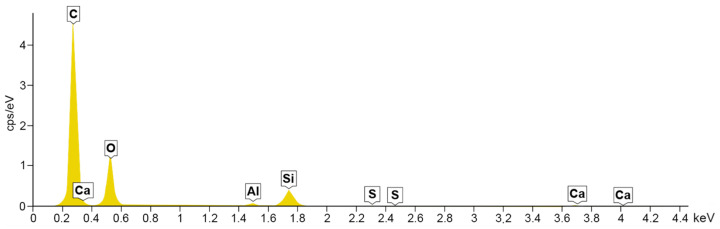
EDS for material M1.

**Figure 10:**
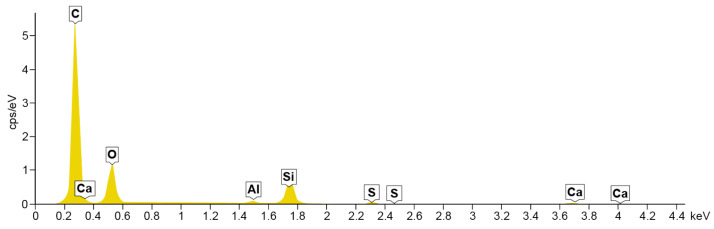
EDS for material M2.

**Figure 11:**
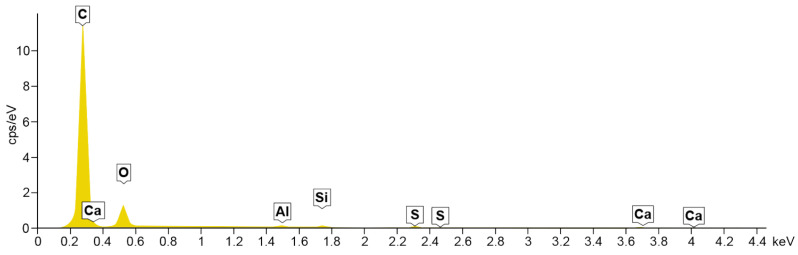
EDS for material M3.

**Figure 12:**
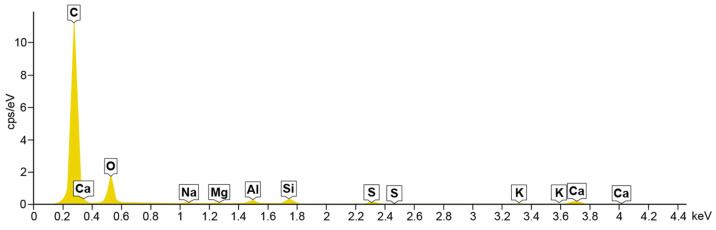
EDS for material M4.

A composition study was also performed for all samples using energy dispersion X-ray spectroscopy. Oxford Ultim Max 65 analyser was used for this purpose. Measurements of the content of chemical elements in the material were made on the surfaces of cross-sections in the middle zones of these sections, i.e., between the bottom and the sample surface. In Figure 9, Figure 10, Figure 11 and Figure 12, charts with chemical compositions of tested materials are presented. Additionally, the chemical composition with crucial chemical elements whose amount exceeds 1% is summarised in Table 1, Table 2, Table 3 and Table 4.

**Figure 13:**
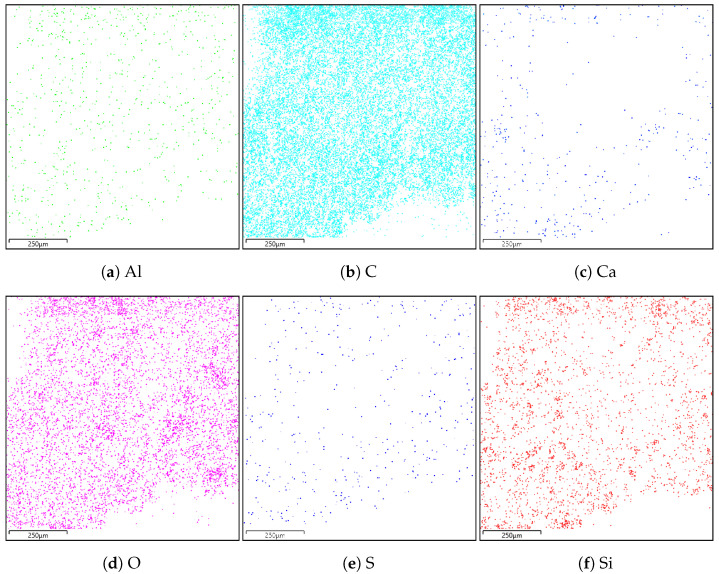
Distribution of individual chemical elements on the cross-sectional area in the material M1.

**Figure 14:**
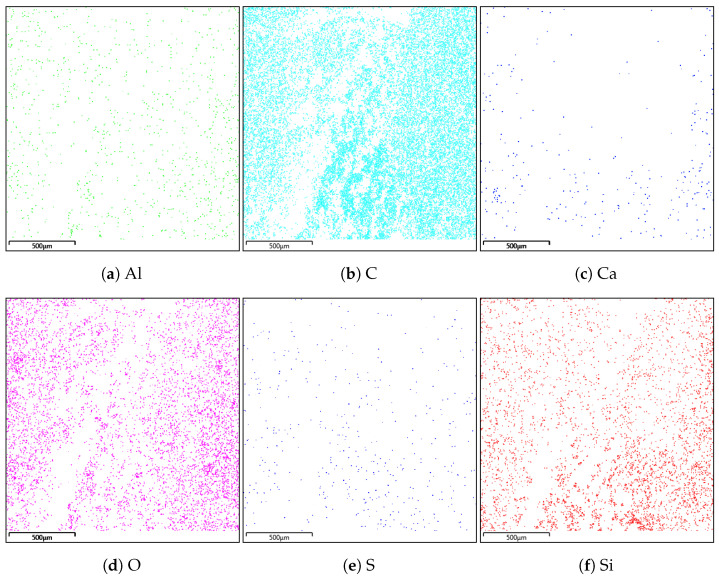
Distribution of individual chemical elements on the cross-sectional area in the material M2.

**Figure 15:**
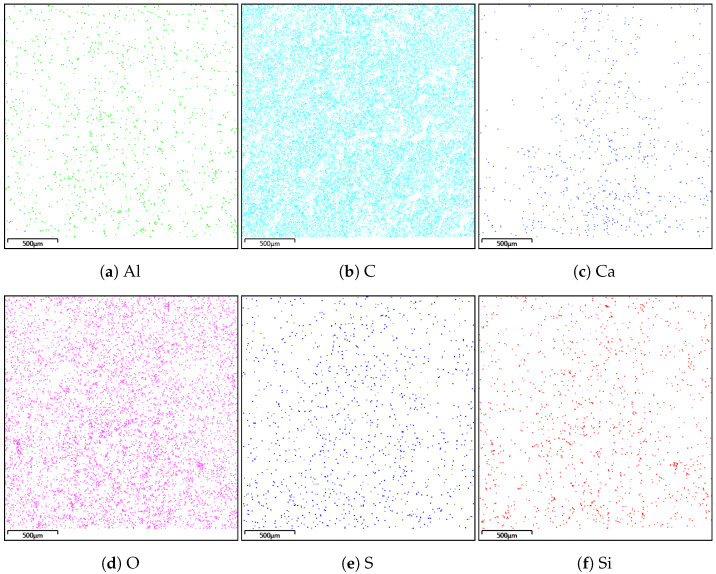
Distribution of individual chemical elements on the cross-sectional area in the material M3.

**Figure 16:**
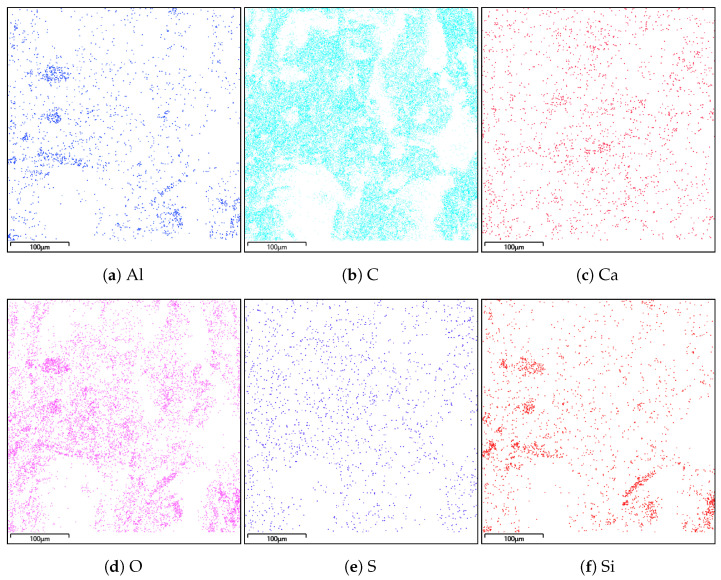
Distribution of individual chemical elements on the cross-sectional area in the material M4.

In all samples, carbon is the most abundant; its content varies around 70%. Oxygen is in second place with an amount of about 20%. Aluminium, silicon occurs in smaller quantities, as well as trace amounts of calcium, magnesium, sulfur and sodium were found. Secondly, in Figure 13, Figure 14, Figure 15 and Figure 16 the distribution of the detected by EDS chemical elements on the sample’s surface is shown. All materials, from M1 to M4, show a uniform distribution of elements in the entire volume of the samples—it was found on the basis of the analysis of the samples carried out on the cross-sectional area. The maps of the primary grid distribution for the M4 material show a relatively high degree of internality. This is equivalent to a large number of individual components on the analysed areas. Hence, the highest density of the M4 material should be expected. Due to the uniform distribution of the elements, no gradient changes in the mechanical properties of the samples are expected, which was proved in the part of this work describing the strength in the mechanical test. The tested roofing materials in the form of felts, also in the macroscopic approach, will show homogeneous mechanical properties.

## 5. Mechanical Test

**Figure 17:**
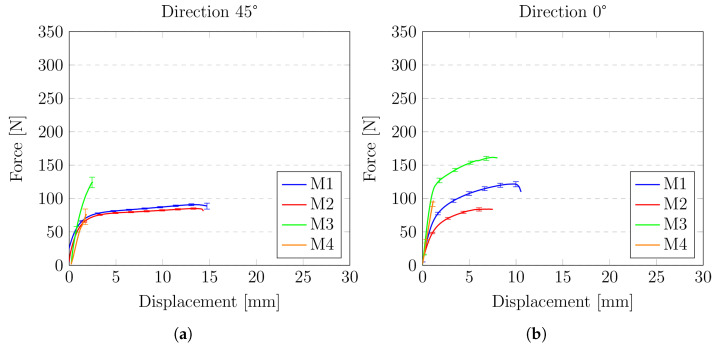

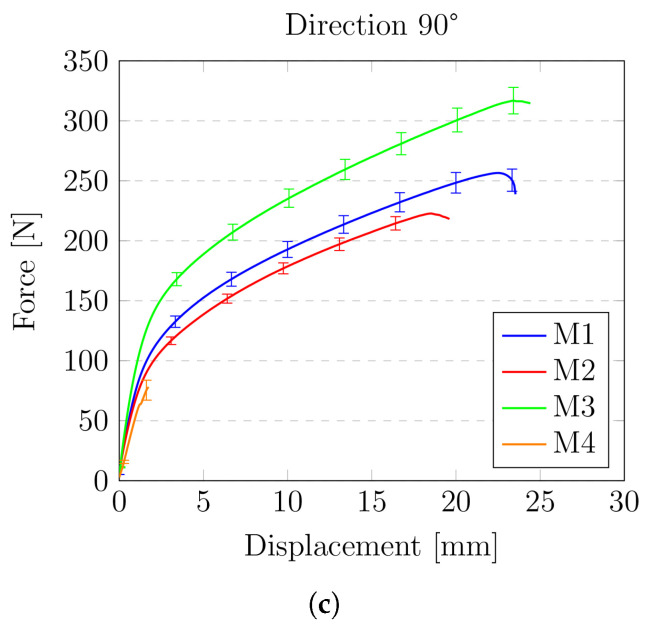
Comparison of the behaviour of materials with regard to the angle of the matrix: (**a**) 45 °; (**b**) 0 °; (**c**) 90 °.

**Figure 18:**
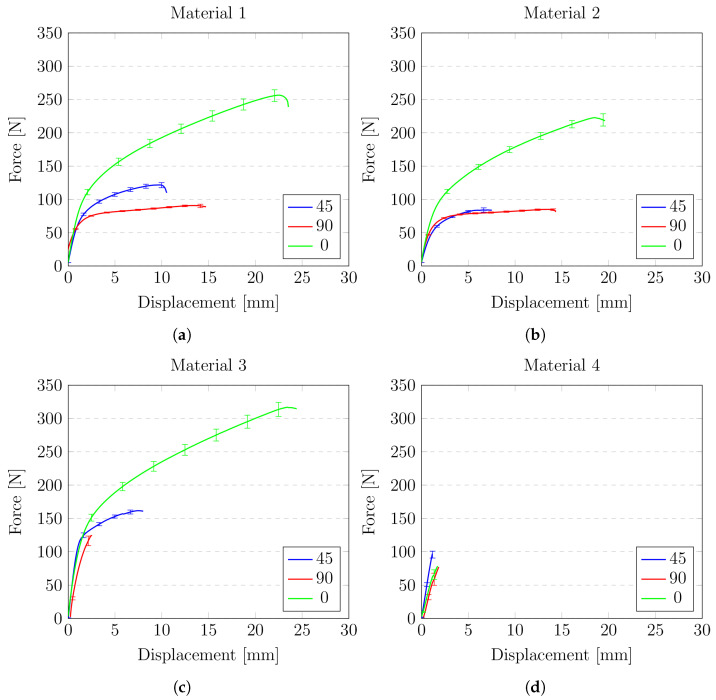
Comparison of mechanical behaviour of tested samples: (**a**) material M1; (**b**) material M2; (**c**) material M3; (**d**) material M4.

The results of the experiments are presented in Figure 17 and Figure 18. Two types of graphs have been prepared. The first type shows a comparison of the analysed materials (M1–M4) for a specific angle at which the specimen was cut with respect to the fibres of the material matrix (0°, 45°, 90°). The second type focuses on the behaviour of one material in relation to the reinforcement direction.

As it was mentioned above, the first three diagrams present the force-displacement curves for all four types of analysed roofing felt (M1, M2, M3, M4) depending on the cutouts of the sample in relation to the material fibres. Presented results were averaged—the spread of a data set is characterised by standard deviation. As it was expected, the highest strength for all of the materials was observed for the direction consistent with the direction of fibres—M1(0°), M2(0°), M3(0°), M4(0°). The most robust material (M3(0°)) reaches above 300 N of the force before it breaks up at the same time; this material was characterised by a relatively significant elongation of 25 mm which is about 35% of sample reference length 70 mm. On the opposite side material, M4(0°) shows the lowest strength and the smallest elongation of 2 mm, which is about 3% of reference length.

Another interesting aspect that can be noticed in the results is the impact of the base material of the sample on the final strength. Material M2 is one of the thinnest of all the analysed (samples made from it has only 2.2 mm thickness); however, this material gives very good strength. The area of the cross-section is over two times smaller than for the highest strength material M3. Going further, if we determine the stresses in cross-section it will be even higher. Material M2 is the only material in which modified bitumen synthetic rubber is used.

The diagrams also clearly show the differences between the reinforcing matrix material. Roofing felts in which the reinforcing matrix was made of non-woven polyester(materials M1, M2, M3) show similar behaviour, whereas, for the material (M4) in which the matrix is a glass veil, the nature of the plot is entirely different

Figure 18a–d are focused on the comparison of the behaviour of a single material. These diagrams show how the direction of the fibres influences the behaviour of the tested material.

For material M1, when loading is applied on 45° or 90° to fibres the behaviour is very similar. The force’s value difference is less than 10%, and elongation is less than 1% (1 mm). Much more difference is when the loading is applied at 0° to the fibres. Then material shows completely different behaviour—the breaking force obtains its maximum value, but there is no elongation. For this case, the elongation of specimen equals only 2.0 mm, which is about 3%.

Material M2 shows more variation in force destroying the sample for the weakest case, compared with the strongest case, reaches over 100 N, and elongation for the case with maximum force is about 18 mm, which is almost 25% higher. However, attention should be paid to the fact that this material (M2) shows the greatest homogeneity as the fibres change the direction the behaviour of the material shows a very similar force-displacement path. Material M2 also has much greater strength than M1, obtaining in his weakest case almost 100 N higher value of breaking force compared to the best case for material M1.

Material M3 is characterised by the average strength and shows the biggest difference between the results obtained for different directions.

Finally, material M4 shows the greatest scatter of behaviour depending on the angle at which the sample was stretched in relation to the matrix. This material was also the material of the lowest strength.

## 6. Summary

The presented research methodology and results are concentrated on the determination of detailed mechanical behaviour of different types of roofing felt. Due to its low cost and relatively simple installation, it is still one of the main materials used as a waterproof insulation in commercial and residential buildings. Therefore, a better knowledge of the material is essential for the further development of the material and new design methods.

Presented results of the static tensile test were prepared for the best scenario i.e., for new material which before tests was kept in ideal conditions. In this case, samples show their greatest strength. Nevertheless, tests showed a clear difference in the mechanical response depending on the material and tested sample. It should be noted that roofing felts are exposed for extreme working conditions which can significantly affect their microscopic structure, which in turn affects strength and durability and what should be included in future research.

Microscopic photos of the material structure and maps of their chemical composition are presented as well. Based on the presented microscopic structure—visible grains of various sizes, no directivity or any kind of structure—it was shown how heterogeneous the felt material is. This heterogeneity affects the mechanical behaviour of the material, which can be observed by the fluctuations of the obtained results for different samples made of the same material. It is even though every single material is produced in modern factory that carefully controls the production process at each stage.

Moreover, the influence of the main matrix reinforcement direction on the strength of materials is discussed. The expected directivity has been demonstrated and is evident in the graphs presented. The presented result also indicates that for each material of this type, minimum three tensile tests in different directions should be performed due to the significant mechanical anisotropy of the material, and that is not possible to infer the behaviour of the material on the basis of a smaller number of tests. It should also be noted that despite the similar geometrical and material properties of the tested materials, the obtained mechanical response is very much dependent on the specific analysed case. It highlights how important the microstructure and direction of the reinforcing fibers are for their strength.

One can conclude that mechanical anisotropy is observed, which is crucial for optimal usage of roofing felt. Furthermore, the discussed results are fundamental from a theoretical point of view, in the sense of strategy used for computational modelling, which is the next step of the presented investigation.

## Figures and Tables

**Table 1:** Chemical composition of material M1.

Element Name	Element Symbol	Amount
Carbon	C	68.5%
Oxygen	O	25.1%
Silicon	Si	4.4%
Calcium	Ca	1.1%
Other	-	0.9%

**Table 2:** Chemical composition of material M2.

Element Name	Element Symbol	Amount
Carbon	C	71%
Oxygen	O	21.6%
Silicon	Si	5.6%
Other	-	1.8%

**Table 3:** Chemical composition of material M3.

Element Name	Element Symbol	Amount
Carbon	C	79.4%
Oxygen	O	17.2%
Calcium	Ca	1.3%
Other	-	2.1%

**Table 4:** Chemical composition of material M4.

Element Name	Element Symbol	Amount
Carbon	C	73.6%
Oxygen	O	19.5%
Calcium	Ca	3.2%
Silicone	Si	1.3%
Other	-	2.4%

## Data Availability

Not applicable.
